# Interpreting changes in life expectancy during temporary mortality shocks

**DOI:** 10.4054/demres.2023.48.1

**Published:** 2023-01-06

**Authors:** Patrick Heuveline

**Affiliations:** 1 California Center for Population Research (CCPR), University of California, Los Angeles (UCLA), Los Angeles, CA 90095.

## Abstract

**BACKGROUND:**

Life expectancy is a pure measure of the mortality conditions faced by a population, unaffected by that population’s age structure. The numerical value of life expectancy also has an intuitive interpretation, conditional on some assumptions, as the expected age at death of an average newborn. This intuitive interpretation gives life expectancy a broad appeal. Changes in life expectancy are also routinely used to assess mortality trends. Interpreting these changes is not straightforward as the assumptions underpinning the intuitive interpretation of life expectancy are no longer valid. This is particularly problematic during mortality ‘shocks,’ such as during wars or pandemics, when mortality changes may be sudden, temporary, and contrary to secular trends.

**OBJECTIVE:**

This study aims to provide an alternative perspective on what changes in life expectancy measure that remains applicable during mortality shocks.

**CONCLUSIONS:**

Returning to two different models that the period life table may represent, I show that a difference in life expectancy is typically interpreted from the synthetic cohort model as the difference in mean longevity between different birth cohorts. However, it can also be interpreted from the stationary population model as a measure of premature mortality in a death cohort. The latter, less common interpretation makes more sense for temporary declines in life expectancy induced by mortality shocks. The absolute change in life expectancy is then an age-standardized value of the average lifespan reduction for people dying during the mortality shock.

**CONTRIBUTION:**

To clarify what a decline in life expectancy measures during mortality shocks is important, especially as demographers often assess the mortality impact of those shocks using this metric, which gets widely reported beyond demographers’ inner circle.

## 1. Introduction

Life expectancy is the most popular mortality indicator with demographers. Unless specified otherwise, it implicitly refers to the value at birth (age 0) of one of the functions derived through a period life table, a key tool of demographic and actuarial analysis. Demographers tend to favor life expectancy because it is a pure measure of the mortality conditions faced by a population during its reference period, unaffected by that population’s age structure. The numerical value of life expectancy also has an intuitive interpretation as the mean age at death of newborns under the assumption that mortality conditions remain as those of the reference period throughout these newborns’ lifetime. If life table construction is limited to an inner circle of demographers and actuaries, this interpretative ease of its numerical value gives life expectancy a much broader appeal.

Demographers also use life expectancy to track mortality trends. As the intuitive interpretation of life expectancy values rests on an assumption of constant mortality, however, the interpretation of year-to-year differences between numerical values of life expectancy is not obvious. Simply put, there would seem to be an inherent contradiction in measuring mortality change with a metric best understood through a thought experiment that assumes constant mortality. A relatively specialized but growing literature has proposed better alternatives to track mortality changes (see Guillot and Payne 2019 for an example and a review). As demonstrated during the COVID-19 pandemic, however, changes in life expectancy remain the most widely reported measures of mortality change. Besides the fact that it remains a pure mortality measure, unaffected by the population’s age structure, one of the reasons to estimate a change in life expectancy is the availability of long time series of life expectancy values. This allows for comparisons of the pace of mortality decline during the pandemic and other mortality shocks – past pandemics, famines, or wars, for example (Aburto et al. 2021; Heuveline 2022). But even demographers sometimes seem to struggle when trying to convey precisely what these declines measure, beyond reflecting changes in a summary mortality statistic or an important measure of population health.

My goal in this article is not to propose a better measure of the pandemic impact on mean longevity but to provide an interpretation for the numerical value of a decline in life expectancy. For example, what does the 1.74-year decline that the United Nations Population Division estimates to be the difference in global life expectancy between 2019 and 2021 (United Nations 2022) actually mean? I argue that rather than a measure of a downward revision in the expected longevity among members of the global population between 2019 and 2021, this numerical value can be better interpreted as a measure of premature mortality among global deaths in 2021. Specifically, the difference in life expectancy is an age standardized value of the mean number of years of life lost in a death cohort, the Mean Unfulfilled Lifespan (MUL), in the same sense that life expectancy is an age standardized value of the Mean Age at Death (MAD) in a death cohort. To establish this, I return to two models of which the period life table might be seen as a representation: a synthetic cohort and a stationary population. The above-mentioned, more common interpretation of life expectancy derives from taking life table functions to represent the hypothetical extinction of a synthetic cohort, an imaginary cohort facing at every age the mortality conditions experienced by population members at that age during the reference period. I then describe how under temporary mortality shocks (i.e., sudden, temporary changes contrary to secular trends), taking the life table functions to represent the characteristics of a stationary population can provide a better understanding of what a decline in life expectancy then measures.

## 2. The period life table as a synthetic cohort

A cohort life table represents the complete survival and eventual extinction of a cohort (typically a birth cohort). In a cohort life table, life expectancy at birth is “the sum of all person-years lived by the cohort divided by the original number in the cohort” (Preston, Heuveline, and Guillot 2001). The number of years lived that each member of the cohort contributes to the sum is their length of life – that is, their age at death. And since in a closed cohort (no migration) the original number in the cohort is the number of deaths, the cohort life expectancy at birth is simply the mean age at death of cohort members. In continuous notation, this can be expressed as

(1) $${\left(e_{0}^{o}\right)}^{C}=\frac{\int_{0}^{\infty }D^{C}(a).ada}{\int_{0}^{\infty }D^{C}(a)da}$$

where ${\left({e_{o}}^{{}^{\circ}}\right)}^{C}$ is the cohort life expectancy at birth and $D^{C}(a)$ represents the number of cohort members dying at age a (and thus living exactly a years) in a closed cohort. Cohort life tables can also represent the projected survival and remaining life expectancy of the current (i.e., not fully extinct yet) cohorts based on the mortality experienced by earlier cohorts (e.g., Goldstein and Lee 2020).

By comparison, a period life table can be seen as a representation of what would happen to a hypothetical (‘synthetic’) cohort if its members were subjected for their entire lives to the mortality conditions of the reference period, which are typically operationalized as a set of age-specific death rates for that period (Preston, Heuveline, and Guillot 2001). In continuous notation, the period life expectancy at birth can similarly be expressed as

(2) $$e_{0}^{o}=\frac{\int_{0}^{\infty }d(a).ada}{\int_{0}^{\infty }d(a)da}$$

where ${e_{0}}^{o}$ is the period life expectancy at birth and d(a) represents the number of decrements at age a in the life table. Starting with an arbitrary number of cohort members at birth, l(0) (the radix of the life table), the construction of the period life table generates the number of survivors to any age a,l(a), resulting from the lifetime exposure to the age-specific mortality rates μ(x) from birth to age a:

(3) $$l(a)=l(0).e^{-\int_{0}^{a}\mu (x)dx}$$

The distribution of deaths, d(a), being obtained by multiplying this distribution of survivors, l(a), by the age-specific death rate at the corresponding age, μ(a), depends only on the radix of the life table and on the age-specific mortality rates μ(a) from birth to a maximum age. As the radix of the life table is a scaling factor that affects the numerator and the denominator of Equation 2 in equal measures, the period life expectancy appears purely as a function of the age-specific mortality rates μ(a). It is independent from other characteristics of the population, such as its age distribution and, in that sense, a pure measure of mortality conditions during the reference period. Its value is the mean age at death among members of the synthetic cohort subjected at each age a to the mortality rate μ(a). In a probabilistic framework, this mean age represents the expected length of life of an average member of the synthetic cohort of newborns subjected throughout their entire lifetime to the mortality rates of the reference period. The very term ‘life expectancy’ is closely tied to this interpretation. As it derives from a thought experiment that follows a hypothetical cohort from birth to death, I thereafter refer to this interpretation as ‘forward-looking.’

The fact that it only depends on the age-specific mortality rates is a desirable property of life expectancy in comparisons of mortality conditions across or within populations, or across periods. In some cases, the difference between two life expectancies can still be interpreted in this forward-looking manner. The difference may represent, for instance, how much longer a person could be expected to live under the mortality conditions experienced by one population, or one subpopulation, compared to those experienced by another population, or subpopulation, were the mortality conditions of these two populations or subpopulations to remain unchanged throughout this person’s lifetime. The difference is then often referred to as the gap in life expectancy resulting from one population or subpopulation experiencing higher mortality than another population or subpopulation (e.g., ethnic/racial gap in life expectancy).

Another example would be the difference between life expectancy before and after a hypothetical medical improvement that would eliminate a cause of death, which is also the basis for Nathan Keyfitz’s (1977) seminal paper on changes in life expectancy. The forward-looking interpretation of the difference in life expectancy becomes more challenging, however, when what separates the mortality conditions being compared is a temporary phenomenon, especially when it reverses secular trends (mortality shock). The difference between pre-pandemic life expectancy and life expectancy during the pandemic, for instance, would then compare a counterfactual expected age at death had the pandemic never occurred (and had mortality from other causes not changed either) with a hypothetical expected age at death if pandemic mortality continues throughout a person’s lifetime. Perhaps not entirely impossible with the periodic emergence of new variants, the latter hypothetical scenario is nonetheless relatively unlikely. Independent from the population’s age distribution, this difference in life expectancy may still provide a useful measure of the mortality impact of the pandemic, but its value can no longer be interpreted as a difference in expected ages at death before and during the pandemic.

## 3. The period life table as a stationary population

Alternatively, the period life table can be seen as representing the characteristics of a hypothetical population: the current population’s ‘stationary equivalent.’ This hypothetical population is the stationary population that would emerge if the current population indefinitely experienced the mortality conditions of the reference period, with no migration and a constant number of births per unit of time. Among these population characteristics, the number of survivors to age , l(a), provides the age distribution of the stationary-equivalent population, while life expectancy at birth provides its mean age at death (MAD) (Preston, Heuveline, and Guillot 2001). This can be seen by considering the MAD in the actual population at a given time:

(4) $$MAD=\frac{\int_{0}^{\infty }D(a).ada}{\int_{0}^{\infty }D(a)da}=\frac{\int_{0}^{\infty }N(a).\mu (a).ada}{\int_{0}^{\infty }N(a).\mu (a)da}$$

where D(a) is the number of persons dying at exact age a and N(a) is the number of persons of exact age a in the population (with reference to time omitted to lighten notations). Substituting for the actual population distribution by age, N(a)/N (where N is the total population size), the age distribution in the stationary-equivalent population, $l(a)/{e_{0}}^{o}$, the MAD in the stationary-equivalent population with the age distribution $l(a)/{e_{0}}^{o}$, and age-specific mortality rates μ(a) indeed equals the period life expectancy shown in Equation 2:

(5) $$\frac{\int_{0}^{\infty }l(a).\mu (a).ada}{\int_{0}^{\infty }l(a).\mu (a)da}=\frac{\int_{0}^{\infty }d(a).ada}{\int_{0}^{\infty }d(a)da}=e_{0}^{o}$$

Life expectancy is thus an age-standardized MAD. This standardization differs from the most common form of standardization, which involves replacing the distribution of the population of interest with a standard distribution borrowed from another population or from a population model unrelated to the population of interest. With the type of standardization that life expectancy entails, by contrast, the age distribution being used, l(a), derives from the population’s own age-specific mortality rates (as can be seen in Equation 3). The two types of standardization can be contrasted as external versus internal standardization, but more importantly both types achieve the goal of removing the influence of actual age distributions when comparing demographic indicators in two populations.

The comparison of internally age-standardized indicators in two populations involves applying potentially different age distributions in the two populations, which for some indicators might be perceived as a disadvantage (Modig, Rau, and Ahlbom 2020). For the MAD, however, internal standardization is immune to an issue that can potentially be encountered with external standardization. If population A has twice the mortality rates of population B at every age, an externally standardized MAD would be the same in the two populations. This is counterintuitive as one would expect an average person to die younger in population A. The intuition is often correct because higher mortality would typically (albeit not necessarily) induce a younger age distribution in population A than in population B. Its age distribution reflects a population’s history of fertility, mortality, and migration, and to obtain a pure measure of the phenomenon of interest, external standardization removes all traces of this demographic history. By contrast, substituting a distribution that depends only on its current mortality levels, the internal standardization performed when estimating life expectancy removes only the MAD’s dependence on fertility and migration but imposes some coherence with current mortality levels. Under these conditions, life expectancy in population A would indeed be lower than in population B.

By comparison with the forward-looking interpretation of life expectancy above, interpretating life expectancy as an age-standardized MAD could be described as ‘backward-looking’ in the sense that it refers to a recent ‘death cohort’ (Riffe, Schöley, and Villavicencio 2017) – that is, past members of the population. Less intuitive than its forward-looking counterpart, this interpretation of life expectancy is uncommon. However, there are several situations in which understanding life expectancy as an internally standardized MAD can usefully complement a forward-looking interpretation. First, this alternative interpretation applies equally well to very short reference periods. When mortality conditions change rapidly, one may want to track these changes with high frequency for periods shorter than a full year (e.g., Trias-Llimos, Riffe, and Bilal 2020; Ghislandi et al. 2022). Due to the seasonality of mortality conditions, however, the forward-looking interpretation of life expectancy for reference periods shorter than a full year implies an implausible lifetime experience, looping conditions in some seasons while skipping conditions in other seasons. Ho and Noymer (2017) refer to such period life expectancies as “pseudo seasonal” expectancies. Second, as described above, the forward-looking interpretation is problematic when considering differences in life expectancy resulting from temporary mortality shocks.

What then is the corresponding interpretation of a difference in life expectancy? Rewriting Pollard’s (1988) formula, the difference in life expectancy appears as follows (see Appendix Part 1 for a derivation of this equation and its relation to Keyfitz’s (1977) hypothetical change mentioned above):

(6) $${\left(e_{0}^{o}\right)}^{B}-{\left(e_{0}^{o}\right)}^{A}=-\frac{\int_{0}^{\infty }\left(\mu^{B}(a)-\mu^{A}(a)\right).l^{B}(a).{\left(e_{a}^{o}\right)}^{A}da}{\int_{0}^{\infty }\mu^{B}(a).l^{B}(a)da}$$

where superscripts A and B refer to two different life tables (corresponding to different populations or subpopulations or to the same population in two different periods). Extending the stationary-equivalent interpretation of the life table, the absolute value of the (negative) difference in life expectancy shown in Equation 6 can be seen as an internally standardized mean unfulfilled lifespan (MUL), a measure of premature mortality:

(7) $$MUL=\frac{\int_{0}^{\infty }\left(\mu^{B}(a)-\mu^{A}(a)\right).N^{B}(a).{\left(e_{a}^{o}\right)}^{A}da}{\int_{0}^{\infty }\mu^{B}(a).N^{B}(a)da}$$

As a measure of premature mortality, the MUL is positive when comparing prevailing mortality conditions to more favorable ones in the past, in which case the difference in life expectancy is negative. The only other difference between the two ratios is that the difference in life expectancy, shown in Equation 6, depends on the distribution of survivors by age in the period life table B, whereas as shown in Equation 7 the actual age distribution of population $B,N^{B}(a)$, is used to calculate the MUL. The original article introducing the MUL provides a practical shortcut for this calculation, with an application to COVID-19 mortality (Heuveline 2021). The rest of the present article aims to provide intuition for interpreting the value of the MUL and comparing it with other measures of premature mortality.

To interpret the values of the MUL and of its stationary equivalent, the difference in life expectancy, first note that when mortality rates at age a are higher in the period life table B than in the period life table A, the first product in the numerator of the MUL represents excess deaths at age $a,D^{E}(a)$. This number is defined as the difference between the actual number of deaths at age a in population or during period $B,\;D^{B}(a)$, and a counterfactual number of deaths in population B had the (typically lower) mortality rate in population or during period A prevailed instead at that age:

(8) $$D^{E}(a)=D^{B}(a)-\left(\mu^{A}(a)\cdot N^{B}(a)\right)=\left(\mu^{B}(a)-\mu^{A}(a)\right)\cdot N^{B}(a)$$

Each excess death at age a corresponds to a person who died at that age in the population or during period B and who would have lived in the population or during period A. The number of additional years this person would have been expected to live is the life expectancy at age a in the population or period $A,{\left({e_{a}}^{o}\right)}^{A}$. This life expectancy thus represents the number of years of life lost by that person due to the mortality differences between the two populations or periods. Summing across all excess deaths yields the numerator of the MUL, a quantity known as years of life lost to a cause of death (Dempsey 1947; Greville 1947; Dickinson and Welker 1948) or, in this case, to excess mortality (i.e., to the difference in mortality between the two populations or periods), $YLL^{E}$:

(9) $$YLL^{E}=\int_{0}^{\infty }D^{E}(a)\cdot {\left(e_{a}^{o}\right)}^{A}da$$

Meanwhile, the sum in the MUL denominator adds up to all the deaths (at all ages) in the population or during period , $D^{B}$.


(10) $$MUL=\frac{YLL^{E}}{D^{B}}$$

Averaging $YLL^{E}$ over the total number of deaths, the MUL. thus represents the average number of years of life lost to excess mortality per death (irrespective of cause of death) in population or during period B.

The MUL can be related to several other indicators of premature mortality (see Appendix Part 2). One of these indicators is “an easily understood measure of excess mortality” (Aron and Muellbauer 2022), the ratio of excess to expected deaths in the absence of mortality changes, the P-score:

(11) $$P=\frac{\int_{0}^{\infty }\left(\mu^{B}(a)-\mu^{A}(a)\right)\cdot N^{B}(a)da}{\int_{0}^{\infty }\mu^{A}(a)\cdot N^{B}(a)da}$$

The MUL can be written as depending on the P-score, on the one hand, and on the average number of years of life lost to excess mortality per excess death, on the other hand:

(12) $$MUL=\frac{P}{1+P}.\frac{\int_{0}^{\infty }D^{E}\left(a\right).{\left(e_{a}^{o}\right)}^{A}da}{D^{E}}$$

Doing so provides intuition for the value of the MUL. This value is the product of (1) the proportion of all deaths in the population or period B that can be considered excess deaths by comparing the prevailing mortality conditions in the population or during period B to the more favorable mortality conditions in the population or during period A (the ratio P/1+P) and (2) how much longer on average a person who died due to the difference in these mortality conditions would have been expected to live under the more favorable conditions in the population or during period A.

As an illustration, Karlinsky and Kobak (2022) estimate that 511,232 of the 3,455,604 deaths in the United States in 2021 (14.8%) are excess deaths that would not have occurred under pre-pandemic mortality conditions. Meanwhile, Goldstein and Lee (2020) estimate that those who died of COVID-19 had on average 11.7 years of remaining life expectancy. This number likely increased in 2021 relative to 2020 as the age structure of COVID-19 mortality shifted toward younger ages as a result of higher vaccination rates among the elderly. If COVID-19 accounted for the vast majority of excess deaths in the United States in 2021 relative to 2019, this suggests the US MUL would be at least 1.73 years in 2021.

Easy to interpret, the value of the MUL depends on demographic characteristics other than mortality, such as the population age distribution. All else equal, the average number of years of life lost to excess mortality per excess deaths takes larger values in populations with younger age distributions because the age distribution of excess deaths itself shifts toward younger ages, at which the values of life expectancy are larger. The value of the P-score also depends on the population age structure (except in special cases, such as when the relative difference in age-specific mortality rates between the prevailing and more favorable conditions is the same at all ages). By substituting the stationary-equivalent distribution to the actual population distribution, the absolute value of the difference in life expectancy then provides a useful internal age-standardization of the value of the MUL, similar to life expectancy with respect to the MAD. To return to the example of the United States, the 2019–2021 decline in life expectancy at birth has been estimated at 2.7 years (Arias et al. 2022). This is substantially larger than the value of the MUL approximated above. This is in part because causes other than COVID-19, with a younger mean age at death, also contributed to excess mortality in 2021 but mostly because the current US age distribution is older than its stationary equivalent – a consequence of the aging of the large birth cohorts of the baby-boom years.

## 4. Conclusion

The most common interpretation of life expectancy has been described above as forward-looking in the sense that it derives from a thought experiment in which members of a birth cohort are hypothetically exposed to unchanging mortality conditions throughout their lifetime. When mortality changes relatively slowly and regularly, differences in life expectancy remain useful, albeit imperfect, indicators of differences in mean longevity. During mortality shocks, however, a decline in life expectancy is hardly interpretable within that framework, and its value may not convey useful information about members of the population who have survived the shock. In such cases, an alternative interpretation described as backward-looking in the sense that it refers instead to a death cohort of past population members has the advantage of remaining interpretable irrespective of the intensity, duration, or direction of mortality changes. The value of the period life expectancy can then be interpreted as an age-standardized MAD and the absolute value of the difference in life expectancy as an age-standardized MUL in a death cohort. The decline in life expectancy is then a measure of premature mortality, whose value is the stationary equivalent of the average lifespan reduction among those who died during a mortality shock.

## Appendix

### Part 1: Keyfitz’s approximation and the actual difference in period life expectancy at birth

To illustrate the impact on life expectancy at birth of a hypothetical medical improvement (assuming no other change in future mortality), Keyfitz (1977) assumes the same relative improvement in age-specific death rates at all ages:

(A1)
$$\frac{\left(\mu^{A}(a)-\mu^{B}(a)\right)}{\mu^{B}(a)}=\delta$$

where superscripts B and A refer to the original (before improvement) and the cause-deleted (after improvement) life table, respectively, and δ is a small negative quantity. After a Taylor expansion, Keyfitz shows that if δ is small enough for $\delta^{2}$ to be considered negligible relative to δ, then

(A2)
$$\frac{{\left(e_{0}^{o}\right)}^{A}-{\left(e_{0}^{o}\right)}^{B}}{{\left(e_{0}^{o}\right)}^{B}}≃\delta .\frac{\int_{0}^{\infty }l^{B}(a).ln\left(l^{B}(a)\right)da}{\int_{0}^{\infty }l^{B}(a)da}=-\delta .H^{B}$$

where $H^{B}$ is the entropy of the original life table. Goldman and Lord (1986) further show that $H^{B}$ can also be written as

(A3)
$$H^{B}=\frac{\int_{0}^{\infty }d^{B}(a).{\left(e_{a}^{o}\right)}^{B}da}{\int_{0}^{\infty }l^{B}(a)da}$$

It follows that a difference in life expectancy can be approximated as

(A4)
$${\left(e_{0}^{o}\right)}^{A}-{\left(e_{0}^{o}\right)}^{B}≃-\delta .H^{B}.{\left(e_{0}^{o}\right)}^{B}\;=-\delta .\frac{\int_{0}^{\infty }d^{B}(a).{\left(e_{a}^{o}\right)}^{B}da}{\int_{0}^{\infty }l^{B}(a)da}.{\left(e_{0}^{o}\right)}^{B}=\;\;=-\delta .\frac{\int_{0}^{\infty }d^{B}(a).{\left(e_{a}^{o}\right)}^{B}da}{\int_{0}^{\infty }d^{B}(a)da}$$

The right side of the equation is the “e-dagger,” a concept described in Vaupel and Canudas Romo (2003):

(A5)
$${\left(e_{0}^{o}\right)}^{A}-{\left(e_{0}^{o}\right)}^{B}≃-\delta .{\left(e_{0}^{o}\right)}^{†B}$$

More generally, Pollard (1988: 266) shows that a difference in life expectancy can be written exactly as

(A6)
$${\left(e_{0}^{o}\right)}^{B}-{\left(e_{0}^{o}\right)}^{A}=\int_{0}^{\infty }\;\left(\mu^{A}(a)-\mu^{B}(a)\right).{}_{a}p_{0}^{B}.{\left(e_{a}^{o}\right)}^{A}da$$

where μ(a) denotes the mortality rate at exact age a, ${}_{a}p_{0}$ as the probability to survive from birth to age a, and ${e_{a}}^{o}$ as the life expectancy at age a. The superscripts A and B refer to two different life tables (corresponding to different populations or subpopulations, or to the same population at two different times). Intuition for Equation 9 can be derived from rewriting the sum as

(A7)
$${\left(e_{0}^{o}\right)}^{B}-{\left(e_{0}^{o}\right)}^{A}=-\frac{\int_{0}^{\infty }\;\;\left(\mu^{B}(a)-\mu^{A}(a)\right).l^{B}(a).{\left(e_{a}^{o}\right)}^{A}da}{\int_{0}^{\infty }\mu^{B}(a).l^{B}(a)da}$$

To tie Pollard’s more general approach to Keyfitz’s approximation, in the case of identical relative changes in mortality rates at all ages expressed in Equation A1, Equation A7 becomes

(A8)
$${\left(e_{0}^{o}\right)}^{B}-{\left(e_{0}^{o}\right)}^{A}=\delta .\frac{\int_{0}^{\infty }\mu^{B}\left(a\right).l^{B}\left(a\right).{\left(e_{a}^{o}\right)}^{A}da}{\int_{0}^{\infty }\mu^{B}\left(a\right).l^{B}(a)da}=\delta .\frac{\int_{0}^{\infty }d^{B}\left(a\right).{\left(e_{a}^{o}\right)}^{A}da}{\int_{0}^{\infty }d^{B}(a)da}$$

As shown in the last ratio, the difference in life expectancy is then proportional to a weighted average of life expectancies after the mortality change, with the weights provided by life table decrements in the original table (before the change). The exact Equation A8 is similar to the approximation in Equation A4. If relative changes in mortality are the same at all ages and relatively small, the approximation in Equation A4 shows that original life expectancies (that include the ‘deleted’ cause of death) can be used instead of the life expectancies after the deletion. (Equation A4 could also be derived directly from Equation A8 through a Taylor expansion.)

### Part 2: The MUL and other indicators of premature mortality

The MUL is an average number of years of life lost to excess mortality, $YLL^{E}$, per death in population B. Averaging instead $YLL^{E}$ over the number of excess deaths, $D^{E}$, would indicate the average number of years of life lost per excess death in population B. To express this, the MUL can be written as

(A9)
$$MUL=\frac{D^{E}}{D^{B}}.\frac{\int_{0}^{\infty }D^{E}\left(a\right).{\left(e_{a}^{o}\right)}^{A}da}{D^{E}}$$

The first ratio expresses the relative incidence of excess and all-cause mortality, frequently measured via the P-score. In the case of identical relative increases in mortality at all ages, the P-score equals the scalar δ in Equation A1, which is then positive. The first ratio in Equation A9 can be written as a function of the P-score:

$$\frac{D^{E}}{D^{B}}=\frac{\int_{0}^{\infty }\left(\mu^{B}(a)-\mu^{A}(a)\right)\cdot N^{B}(a)da}{\int_{0}^{\infty }\mu^{B}(a)\cdot N^{B}(a)da}=\frac{\int_{0}^{\infty }\left(\mu^{B}(a)-\mu^{A}(a)\right)\cdot N^{B}(a)da}{\int_{0}^{\infty }\mu^{A}(a)\cdot N^{B}(a)da}\cdot \frac{\int_{0}^{\infty }\mu^{A}(a)\cdot N^{B}(a)da}{\int_{0}^{\infty }\mu^{B}(a)\cdot N^{B}(a)da}=\frac{P}{1+P}.$$

The MUL can then be expressed as

(A10)
$$MUL=\frac{P}{1+P}.\frac{\int_{0}^{\infty }D^{E}\left(a\right).{\left(e_{a}^{o}\right)}^{A}da}{D^{E}}$$

In the literature on premature mortality, $YLL^{E}$ is often related to the total size of the population instead. To express this, the MUL can also be written as

(A11)
$$MUL=\frac{N^{B}}{D^{B}}.\frac{\int_{0}^{\infty }D^{E}(a).{\left(e_{a}^{o}\right)}^{A}da}{N^{B}}=\frac{1}{CDR^{B}}.\frac{\int_{0}^{\infty }D^{E}(a).{\left(e_{a}^{o}\right)}^{A}da}{N^{B}}$$

where $CDR^{B}$ is the crude death rate and $N^{B}$ is the population size in population B.

Both terms of the product in Equation A11 can be standardized using an ‘external’ standard population distribution, $N^{S}(a)$. After standardization, the first ratio would become the inverse of the classic age-standardized crude death rate, ASCDR, in population B (Preston, Heuveline, and Guillot 2001):

(A12)
$$ASCDR^{B}=\frac{\int_{0}^{\infty }N^{S}\left(a\right).\mu^{B}(a)da}{\int_{0}^{\infty }N^{S}(a)da}$$

The second ratio can be written as

(A13)
$$\frac{\int_{0}^{\infty }D^{E}\left(a\right).{\left(e_{a}^{o}\right)}^{A}da}{N^{B}}=\frac{\int_{0}^{\infty }N^{B}(a).\left(\mu^{B}(a)-\mu^{A}(a)\right).{\left(e_{a}^{o}\right)}^{A}da}{\int_{0}^{\infty }N^{B}(a)da}$$

It appears as a weighted average of the $YLL^{E}$ rates at age a (Martinez et al. 2019), $YLLR^{E}(a)$:

(A14)
$$YLLR^{E}(a)\;=\frac{YLL^{E}(a)}{N^{B}(a)}=\frac{D^{E}(a).{\left(e_{a}^{o}\right)}^{A}}{N^{B}(a)}=\frac{\left(\mu^{B}(a)-\mu^{A}(a)\right).N^{B}(a).{\left(e_{a}^{o}\right)}^{A}}{N^{B}(a)}=\;\;=\left(\mu^{B}(a)-\mu^{A}(a)\right).{\left(e_{a}^{o}\right)}^{A}$$

In Equation A13, the weights are provided by the population distribution. If we substitute instead the standard population distribution, we get the age-standardized $YLL^{E}$ rate, $ASYR^{E}$:

(A15)
$$ASYR^{E}=\frac{\int_{0}^{\infty }N^{S}(a).YLLR^{E}(a)da}{\int_{0}^{\infty }N^{S}(a)da}$$

The ratio $ASYR^{E}/ASCDR^{B}$ is thus an externally standardized MUL, whereas the absolute value of the difference in life expectancy is an internally standardized MUL.
